# Phase II trial of S‐1 treatment as palliative‐intent chemotherapy for previously treated advanced thymic carcinoma

**DOI:** 10.1002/cam4.3385

**Published:** 2020-08-19

**Authors:** Yusuke Okuma, Yasushi Goto, Fumiyoshi Ohyanagi, Kuniko Sunami, Yoshiro Nakahara, Satoru Kitazono, Keita Kudo, Yuichi Tambo, Shintaro Kanda, Noriko Yanagitani, Atsushi Horiike, Hidehito Horinouchi, Yutaka Fujiwara, Hiroshi Nokihara, Noboru Yamamoto, Makoto Nishio, Yuichiro Ohe, Yukio Hosomi

**Affiliations:** ^1^ Department of Thoracic Oncology and Respiratory Medicine Tokyo Metropolitan Cancer and Infectious Diseases Center Komagome Hospital Tokyo Japan; ^2^ Department of Thoracic Oncology National Cancer Center Hospital Tokyo Japan; ^3^ Department of Thoracic Medical Oncology The Cancer Institute Hospital of Japanese Foundation for Cancer Research Tokyo Japan; ^4^ Division of Pulmonary Medicine Clinical Department of Internal Medicine Jichi Medical University Saitama Medical Center Saitama Japan; ^5^ Department of Pathology and Clinical Laboratories National Cancer Center Hospital Tokyo Japan; ^6^ Department of Thoracic Oncology Kanagawa Cancer Center Yokohama Japan; ^7^ Department of Thoracic Medical Oncology National Hospital Organization Osaka Minami Medical Center Osaka Japan; ^8^ Respiratory Medicine Kanazawa University Ishikawa Japan; ^9^ Division of Medical Oncology Department of Medicine Showa University School of Medicine Tokyo Japan; ^10^ Department of Respiratory Medicine Mitsui Memorial Hospital Tokyo Japan; ^11^ Department of Respiratory Medicine and Rheumatology Graduate School of Biomedical Sciences Tokushima University Tokushima Japan

**Keywords:** chemotherapy, phase II, rare cancer, S‐1, thymic carcinoma

## Abstract

Thymic carcinoma (TC) is a rare cancer with minimal evidence of survival following palliative‐intent chemotherapy. Sunitinib, everolimus, and pembrolizumab have been proposed as active agents based on previous phase II trials. In this phase II study, TC patients previously treated with platinum‐based chemotherapy were enrolled. The patients received S‐1 orally twice daily at a dose of 40‐60 mg/m^2^ for 4 weeks, followed by 2 weeks off until the progression of the disease or the presence of unacceptable toxicities. The primary endpoint was the objective response rate (ORR), and secondary endpoints were progression‐free survival (PFS), overall survival (OS), and safety. The sample size of 26 patients was planned to reject the ORR of 10% under the expectation of 30% with a power of 0.80 and a type I error of 0.05 (one‐sided). Twenty‐six patients were recruited between 2013 and 2016; 23 patients had squamous cell carcinoma and 10 had an ECOG performance status of 0. One patient showed complete response and seven patients showed partial responses, resulting in a 30.8% response rate (90% confidence interval [CI], 18.3‐46.9) and an 80.8% disease control rate (90% CI, 65.4‐90.3). The median PFS was 4.3 months (95% CI, 2.3‐10.3 months) and median OS was 27.4 months (95% CI, 16.6‐34.3). Adverse events of grade ≥ 3 included neutropenia (12%), skin rash (8%), elevated alanine aminotransferase, and fatigue (4%). No treatment‐related death was observed. S‐1 confirmed clinical activity with tolerability in patients with previously treated TC. (UMIN000010736).

## Graphical abstract

Not a few patients of TC can be treated with S‐1 in a longer period. The toxicity of S‐1 was also acceptable. S‐1 is a promising drug for TC with easy accessibility and low cost.

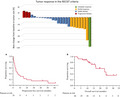

## 1 INTRODUCTION

Rare cancers are one of the reasons for the delay in improvements in cancer treatment owing to the unfeasibility of large clinical trials. The RARECARE project^1^ supported by the European Commission focuses on rare cancers to overcome these issues because they collectively represent about 22% of all cancers, despite the rarity of each subtype among 186 rare cancers.^2^ Based on the RARECARE definition, rare cancers have an annual incidence of less than six per 100 000 persons.^3^ Additionally, Rare Cancers Europe recently published a consensus position paper for clinical trials on rare cancers.^4^

Thymic malignancies comprising thymomas and thymic carcinomas are rare cancers according to the definition provided by RARECARE; the incidence of thymic epithelial tumors is 0.15‐0.32 cases per million and thymic carcinomas account for approximately 10%‐15% of them.^5^ According to the 2016 World Health Organization (WHO) classification, thymic carcinoma is divided into thymoma based on the clinical and immunological complications or the aggressiveness of the tumor. Fundamentally, immunological complications are not demonstrated in patients having thymic carcinoma with loss of thymic function. Thymic carcinoma has a poor prognosis compared to thymomas with a 5‐year survival rate of 30%‐50%.^6^, ^7^, ^8^ Patients with metastatic thymic carcinoma are treated with palliate‐intent chemotherapy or supportive care. Nevertheless, optimal chemotherapy has not been determined because of the rarity of this disease. Platinum combination chemotherapy with or without anthracycline is a widely used first‐line treatment method for thymic carcinoma, with response rates ranging from 20% to 50%.^9^, ^10^ There is no confirmatory survival benefit for previously treated thymic carcinoma; however, the use of single‐agent cytotoxic or molecular‐targeted agents has been recommended in the National Comprehensive Cancer Network (NCCN) guidelines.^11^ Sunitinib,^12^ everolimus,^13^ and pembrolizumab^14^, ^15^ showed verified activities in a phase II study from the United States and Europe. S‐1 was anticipated in previously treated thymic carcinoma based on a retrospective study from Japan^16^ with a view of similar efficacy for the aforementioned‐targeted therapies.

S‐1 is one of the key drugs used for gastrointestinal and non‐small cell lung cancers.^17^ The correlation between clinical outcome and the expression of thymidine synthase, dihydropyrimidine dehydrogenase, and orotate phosphoribosyltransferase, to which associated S‐1 activities in thymic carcinomas treated with S‐1 remains unclear.^16^ Among the fluoropyrimidine agents, capecitabine demonstrated clinical activity in thymic carcinoma in combination with gemcitabine, albeit in few patients (N = 8)^18^; gemcitabine may be used as the single agent.^19^ Cytotoxic therapy, including S‐1, has shown similar efficacy in previously treated thymic carcinomas. Moreover, this therapy is cost‐effective compared to other targeted therapies.

Based on retrospective data, we conducted a phase II trial of S‐1 for previously treated thymic carcinomas in the various cancer centers in Tokyo. This study was registered with the UMIN Clinical Trials Registry (UMIN000010736).

## 2 PATIENTS AND METHODS

### 2.1 Patients

This open‐label, single‐arm, phase II trial was conducted at National Cancer Center Hospital, Tokyo Metropolitan Cancer and Infectious Disease Center Komagome Hospital, and The Cancer Institute Hospital of Japan Foundation for Cancer Research. The study protocol was approved by the institutional review boards of all institutions (Clinical trial registration: UMIN000010736). The present study was conducted in accordance with the Declaration of Helsinki and the Guidelines for Good Clinical Practice in Japan. Written informed consent was obtained from all participating patients. S‐1 was used off‐label.

The following eligibility criteria were used: treated with first‐line platinum‐containing chemotherapy; more than 20 years of age; and a histologically or cytologically confirmed previously carcinoma treated with platinum‐containing chemotherapy or Masaoka‐Koga Stages IVa and IVb tumors^20^; measurable lesions as defined by the Response Evaluation Criteria in Solid Tumors (RECIST) 1.1^21^; an Eastern Cooperative Oncology Group (ECOG) performance status (PS) of 0‐2; adequate bone marrow reserve (with leukocyte count equal to or more than 2000 cells/μL; hemoglobin levels equal to or more than 8.5 g/dL; and platelet count equal to or more than 100 000 cells/μL); aspartate aminotransferase (AST)/alanine aminotransferase (ALT) equal to or less than 2.5 times of the upper limit of each hospital and serum bilirubin ≤ 1.5 mg/dL; creatinine level equal to or less than 1.5 mg/dL; and SpO_2_ ≥ 92%.

### 2.2 Treatment

The starting dose of S‐1 was determined according to the body surface area (BSA) as follows: 80 mg daily for BSA < 1.25 m^2^; 100 mg daily for 1.25 ≤ BSA <1.5 m^2^; and 120 mg daily for BSA ≥ 1.5 m^2^. The drug was taken twice daily for 4 weeks, followed by 2 weeks off for a cycle. In cases with poor PS, mild organ impairment, or other reasons suggesting intolerability, the dose was decreased in a stepwise manner or was given for 2 weeks, followed by a 1‐week drug‐free interval per cycle. Treatment continued until disease progression or intolerable toxicity.

### 2.3 Evaluation

The clinical benefits were evaluated using the following parameters: overall response rate (ORR), disease control rate (DCR), progression‐free survival (PFS), and overall survival (OS). We assessed the treatment efficacy of S‐1 using RECIST version 1.1. Disease control rate was defined as an objective responder and stable disease. PFS was calculated from the day of registry until the date of confirmed progression, early discontinuation of treatment or death from any cause, and was censored at the date of the last follow‐up visit for patients who were still alive and who had not progressed. OS was defined as the interval between the date of the registry to the time of death from any cause or the last follow‐up evaluation. Treatment evaluation was assessed using computed tomography or magnetic resonance imaging every 6‐8 weeks. Patients who were alive on the date of the last follow‐up were censored on that date. The external radiographic assessment was reviewed by Lisit Co., Ltd. for the academic research organization.

Toxicity was evaluated using the Common Terminology Criteria for Adverse Events version 4.1 (CTCAE v4.1). The patients were assessed after at least two cycles of chemotherapy, and the duration of response was reported from the date of the first cycle to confirmation of disease progression.

### 2.4 Pathological diagnosis

This study included another extramural review committee of independent pathologists to aid with the definitive diagnosis of thymic carcinoma. The histological type was determined based on the agreement of two of three pathologists. A diagnosis was made using the hematoxylin‐eosin (H&E) staining method and immunohistochemistry (IHC; for CD5, CD117, and synaptophysin) on paraffin‐embedded sections obtained from formalin‐fixed specimens. Information about the expression levels of other proteins was also utilized.

### 2.5 Statistical analysis

The primary endpoint was the ORR, and secondary endpoints were PFS, OS, and safety. The sample size was calculated with a type I error of 0.05 (one‐sided) and a power of 0.80. The activity of S‐1 was considered nonsignificant if the ORR was lower than 10% (null hypothesis based on historical data) and the expectation was considered as promising if the ORR was higher than 30% (alternative hypothesis based on retrospective data). The target sample size was 24 patients after deducting two patients who could not be evaluated from the total of 26 patients. The ORR and DCR were presented with 90% exact binomial confidence intervals (CIs). PFS and OS were estimated using the Kaplan‐Meier method. All statistical analyses were carried out using JMP 11 (SAS Institute Inc).

## 3 RESULTS

### 3.1 Patient characteristics

Twenty‐six patients were enrolled from three cancer centers in the Tokyo metropolitan region of Japan from November 2013 to May 2016. The patient demographics and disease characteristics are summarized in Table 1. Of the 26 patients, 10 (38.5%) were males with a median age of 63 years (range: 27‐74), eight patients (30.8%) demonstrated recurrence with curative‐intent therapy, four demonstrated stage IVa tumors (15.4%), 14 had stage IVb tumors (53.8%), and 10 patients (38.5%) had an ECOG PS of 0. Previous lines of chemotherapy included an average of two lines.

**TABLE 1:** Demographic and baseline patient characteristics

Characteristics	N° of patients (N = 26)	%
Gender		
Male/Female	10/16	38.5/61.5
Age, median (range)/y	63 (27‐74)	
Performance status (ECOG)		
0/1	10/16	38.5/61.5
Masaoka‐Koga stages		
IVa/IVb/Recurrence	4/14/8	15.4/53.8/30.8
Smoking history		
Never/previous or current	9/17	34.6/65.4
Metastatic sites (overlapped)		
Lung	5	
Liver	6	
Lymph nodes	4	
Pleura	5	
Pericardial	1	
Bone	7	
Brain	2	
Histological subtype (institutional diagnosis)		
Squamous cell carcinoma/Borderline/Unknown	23/1/2	88.5/3.8/7.7
Previous chemotherapy		
ADOC	1	3.8
Cisplatin‐irinotecan	3	11.5
Cisplatin‐etoposide	2	7.7
Carboplatin‐paclitaxel	20	77.0
Previous history of radiotherapy		
Yes/No	10/16	38.5/61.5

Abbreviations: ADOC, adriamycin, cisplatin, vincristine, and cyclophosphamide; ECOG PS, Eastern Cooperative Oncology Group Performance Status; No, number.

### 3.2 Pathological diagnosis

In each institution, the pathological diagnosis during enrolment reported that 23 patients had squamous cell carcinoma (88.5%), one patient demonstrated borderline tumors, and two patients had unknown histological subtypes (Table 1). Twenty‐one patients were evaluable for pathological diagnosis by the extramural review committee. The independent review for histological subtype determined that all patients had thymic carcinoma. With regard to the histological subtype, there were 20 squamous cell carcinomas and one basaloid carcinoma.

### 3.3 Treatment delivery and efficacy

**FIGURE 1:**
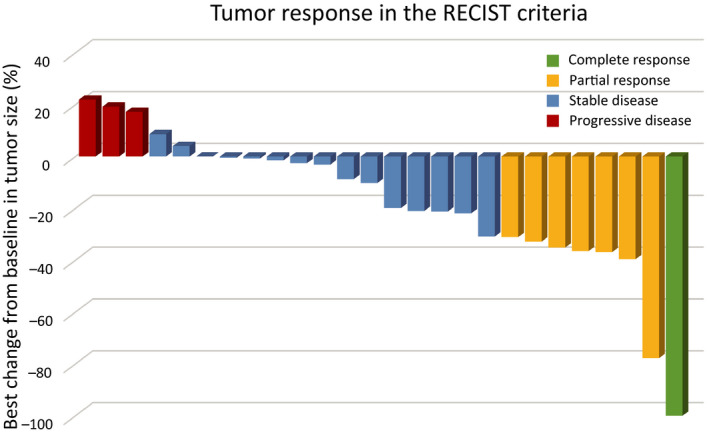
Waterfall plot showing the best response of targeted lesions to S‐1 treatment according to the Response Evaluation Criteria in Solid Tumors

**FIGURE 2:**
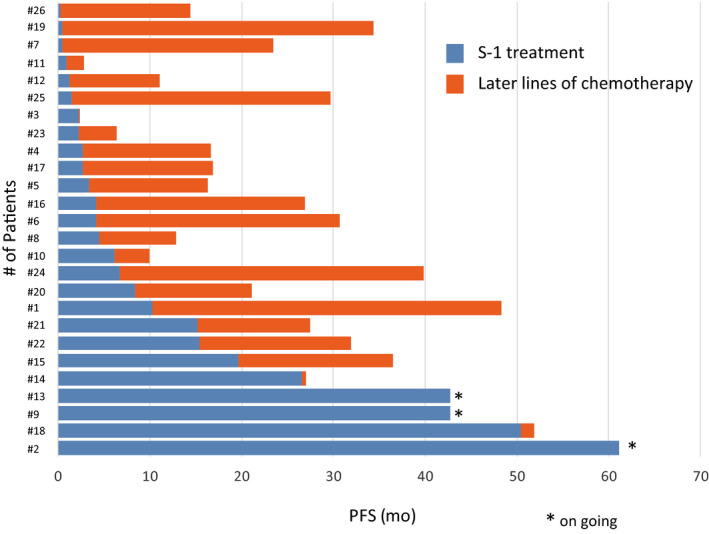
Swimmer's plot of individual patients who were treated with S‐1

**FIGURE 3:**
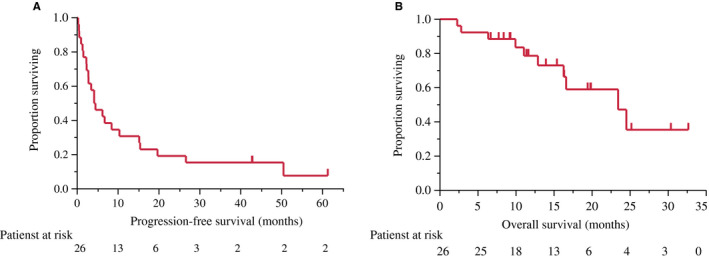
The estimated Kaplan‐Meier curves for (A) progression‐free survival (PFS) and (B) overall survival (OS)

Investigator assessments of the 26 evaluable patients showed that one patient (3.8%) had achieved a complete response (CR) and seven patients (26.9%) had achieved partial response (PR), resulting in a response rate of 30.8% (90% CI, 18.3‐46.9) and a disease control rate of 80.8% (90% CI, 65.4‐90.3; Figure 1). Two patients could not be evaluated because of their progression. The median PFS was 4.3 months (95% CI, 2.3‐10.3 months) and median OS was 27.4 months (95% CI, 16.6‐34.3) after a median follow‐up of 27.0 months (Table 2). The OS rates at 1 and 2 years were 76.9% and 52.6%, respectively (Figures 2 and 3).

**TABLE 2:** Clinical outcomes and Safety profiles of S‐1 treatment for previously treated thymic carcinoma

Clinical outcome	No of patients (N = 26)	
Response rate (CR + PR) (%) [90% CI]	30.8%	[18.3‐46.9]
Disease control rate (CR + PR+SD) (%) [90% CI]	80.8%	[65.4‐90.3]
Median response duration, months [95% CI]	4.3 mo	[2.3‐10.3]
Median overall survival, months [95% CI]	27.4 mo	[16.6‐34.3]
1‐y survival rate, %	76.9%	

Safety profile					
	All grades (%)	Grade 1 (%)	Grade 2 (%)	Grade 3 (%)	Grade 4 (%)
Hematological adverse events					
White blood cell decreased	8 (30.8)	3 (11.5)	2 (7.7)	3 (11.5)	0 (0)
Neutrophil count decreased	10 (38.4)	4 (15.4)	5 (19.2)	1 (3.8)	0 (0)
Anemia	4 (15.4)	3 (11.5)	1 (3.8)	0 (0)	0 (0)
Platelet count decreased	5 (19.2)	5 (19.2)	0 (0)	0 (0)	0 (0)
Nonhematological adverse events					
Diarrhea	11 (42.3)	10 (38.5)	1 (3.8)	0 (0)	0 (0)
Nausea	8 (30.8)	4 (15.4)	4 (15.4)	0 (0)	0 (0)
Aspartate aminotransferase increased	8 (30.8)	7 (26.9)	1 (3.8)	0 (0)	0 (0)
Skin rash	8 (30.8)	3 (11.5)	2 (7.7)	3 (11.5)	0 (0)
Fatigue	6 (23.1)	4 (15.4)	1 (3.8)	1 (3.8)	0 (0)
Anorexia	6 (23.1)	4 (15.4)	2 (7.7)	0 (0)	0 (0)
Alanine aminotransferase increased	4 (15.4)	3 (11.5)	0 (0)	1 (3.8)	0 (0)
Corneal ulcer	0 (0)	1 (3.8)	0 (0)	0 (0)	0 (0)
Febrile neutropenia	0 (0)	0 (0)	0 (0)	0 (0)	0 (0)

Abbreviation: CI, confidence interval.

### 3.4 Adverse events

The hematological and nonhematological toxicities of all patients are summarized in Table 2. The major adverse events were grade 3‐4 decreased leukocytopenia in three (11.5%) patients and skin rash in three patients (11.5%), respectively. Grade 3 neutropenia, fatigue, and increased alanine aminotransferase were observed in one patient (3.8%). Skin rash led to treatment discontinuation before disease progression. No treatment‐related death was observed.

## 4 DISCUSSION

This study demonstrates the use of S‐1 as one of the active drugs for patients with previously treated thymic carcinoma. The active drugs for thymic carcinoma and thymoma may be different considering the heterogeneity of thymic carcinomas.^10^ Sunitinib, everolimus, pembrolizumab, which target c‐kit, serine‐threonine kinase mammalian target of rapamycin (mTOR), and PD‐L1, respectively, demonstrate similar response rates (20%) and PFS (4‐5 months) for previously treated thymic carcinomas (Table 3), whereas cixutumumab targeting for IGF1R is for previously treated thymoma.^22^

**TABLE 3:** Chemotherapy and molecular‐targeted agents for the patients previously tread with thymic carcinoma

Authors	Agent	Study design	Target	n	Response Rate (DCR)	PFS (mo)	OS (mo)
Cytotoxic agents							
Loehrer et al^29^	Pemetrexed	Ph II	—	11	9.1%	2.9	9.8
Wakelee et al^30^	Amrubicin	Ph II	—	19	10.5%	8.5	18.1
Palmieri et al^18^	Capecitabine + gemcitabine	Ph II	—	8	37.5%	6	N/A
Tsukita et al^25^	S‐1	Ph II	—	20	25%	5.4	22.7
Present study	S‐1	Ph II	—	26	30.8% (80.8%)	4.3	27.4
Molecular‐targeted agents							
Thomas et al^12^	Sunitinib	Ph II	c‐KIT, PDGFR	23	26% (65%)	7.2	Not reached
Zucali et al^13^	Everolimus	Ph II	mTOR	12	25% (41%)	12.1^a^	24.0^a^
Rajan et al^22^	Cixutumumab	Ph II	IGF‐1R	12	0%	1.7	8.4
Giaccone et al^31^	Belinostat	Ph II	HDAC	16	0% (50%)	5.8	12.4
Besse et al^32^	Milciclib (PHA‐848125AC)	Ph II	CDK, src family	26	4.2%	9.76	Not reached
Bedano et al^33^	Erlotinib + bevacizumab	Ph II	EGFR, VEGF	7	0	N/A	N/A
Kurup et al^34^	Gefitinib	Ph II	EGFR	7	0	N/A	N/A
Giaccone et al^35^	Imatinib	Ph II	c‐KIT mutation	5	0	N/A	N/A
Loehrer et al^36^	Octreotide + prednisone	Ph II	somatostatin receptor	6	0	4.5	23.4
Gubens et al^37^	Saracatinib (AZD0530)	Ph II	src family	9	0	3.6	6.7
Itoh et al^26^	Lenvatinib	Ph II	VEGFR, FGFR, RET, c‐Kit etc	42	38.1%	9.3	Not reached
Immune check point inhibitors							
Cho et al^14^	Pembrolizumab	Ph II	PD‐1	26	19.2% (73%)	6.1	N/A
Giaccone et al^15^	Pembrolizumab	Ph II	PD‐1	40	22.5% (75%)	4.2	24.9
Katsuya et al^38^	Nivolumab	Ph II	PD‐1	15	0% (73.3%)	3.8	N/A

Abbreviations: CDK, cyclin‐dependent kinase; DCR, disease control rate; epidermal growth factor; HDAC, histone deacetylase; IGF‐1R, insulin‐like growth factor 1 receptor; mTOR, mammalian target of rapamycin; n, number; OS, overall survival; PDGFR, platelet‐derived growth factor; PD‐L1, programmed death‐1; PFS, progression‐free survival; Ph II, phase II; Retrosp, retrospective; VEGF, vascular endothelial growth factor.

^a^ Survival data include thymoma and thymic carcinoma.

The clinical activity of S‐1 has been demonstrated in a small number of patients.^11^ Another fluoropyrimidine, capecitabine, is regarded as an active drug for thymic malignancies.^18^ However, a suitable biomarker for S‐1 was not identified using metabolic enzymes, which play key roles in the metabolism of fluoropyrimidines.^16^ In a multi‐institutional retrospective study comprising 286 patients with thymic carcinoma,^23^ a 20%‐40% response rate was observed with a PFS of 4‐6 months. There are no studies on antimetabolites because of the cytotoxic nature of the agent. However, one question raised that S‐1, gemcitabine, and capecitabine were responded to previously treated thymic carcinoma, whereas pemetrexed had not shown enough in the antimetabolites.^24^ Among them, only S‐1 showed clinical efficacy in a Japanese trial (NJLCSG 1203) comprising 40 patients with invasive thymoma and thymic carcinoma. In the NJLCSG 1203 study, the primary endpoint was not met because of the low power of the study or the weak activity of thymoma.^25^ It is recommended to conduct clinical trials with thymic carcinoma and thymoma with the inclusion of the extramural review committee of independent pathologists to aid with the definitive diagnosis owing to the apparently different biological backgrounds.

The clinical benefit of this treatment remains uncertain compared to supportive care for previously treated thymic malignancies because of the rare nature of cancer. The NCCN guidelines recommend single‐agent or nonplatinum‐based chemotherapy,^11^ but do not provide separate recommendations for thymic carcinoma and thymoma. In the precision medicine era, the molecular investigations and clinical outcomes of thymic malignancies are regarded as important. Recent trials with molecular‐targeted agents and immunotherapy did not meet expectations with regard to toxicity and cost‐effectiveness; severe immunological‐related toxicities, including myocarditis and pneumonitis, were observed with pembrolizumab^14^, ^15^ and everolimus.^13^ A recent study of lenvatinib for thymic carcinoma demonstrated good response (38.1%, 90% CI = 25.6‐52.0) with little PD (4.8%) and the median duration of response of 11.6 months.^26^ There is no biomarker of lenvatinib for thymic carcinoma; however, the result is promising that multikinase inhibitor including antiangiogenic agents is a crucial drug.

Interestingly, the cost benefits of these agents vary. Based on the results of the median PFS from the phase II trials of sunitinib, everolimus, and the present retrospective study for thymic carcinoma, 7.2 months of sunitinib treatment costs about 30 000 EUR (5700 EUR per cycle [6 weeks]), 12.1 months of everolimus treatment costs 68 500 EUR (7300 EUR per 6 weeks), 11.6 months of lenvatinib treatment costs 78 540 EUR (10 155 EUR per 6 weeks), and 8.1 months of S‐1 treatment costs 4200 EUR (700 EUR per cycle [6 weeks]). Cytotoxic agents should be used in clinical trials or observational studies because the response rates in the second, third, and fourth lines of cytotoxic chemotherapy were decreased to 39.1%, 23.1%, and 12.5%, respectively.^27^

The major limitations of the present study were its single‐arm nature and the small sample size. Moreover, the methodology of biological plausibility why S‐1 was effective for thymic carcinoma is lacking because of no biomarker for S‐1. There is no reported actionable biomarker in thymic carcinoma. This remains “no man's land” in thymic malignancies. Due to the small numbers of specimens in metastatic thymic malignancies examined at initial diagnosis, biomarker analysis is hampered. We could centrally be reviewed by pathologists to make a precise pathological diagnosis in the current study, which is often difficult to achieve in the case of multicenter trials. It is important to distinguish between thymic carcinoma and thymomas while selecting active drugs for treatment.^10^ Fatal toxicities have been reported in patients with thymoma who were treated with pembrolizumab^15^, ^28^

Further studies investigating new drugs or combinations of active drugs are essential. Also, additional biological studies on drug development for rare cancers are important.

## 5 CONCLUSIONS

In this phase II study, 26 patients with previously treated thymic carcinoma were treated with single‐agent S‐1 activity against advanced thymic carcinoma in a palliative capacity. New drug candidates for thymic malignancies are increasing, however, the biological plausibility for these drugs need further investigation as well as the evaluation of cost‐effectiveness benefit.

## DISCLOSURE STATEMENT

Dr Yusuke Okuma has received personal fees as honoraria from honoraria from AstraZeneca, Boehringer Ingelheim, Bristol‐Myers Squibb, Chugai, and Ono. Dr Yasushi Goto has received personal fees as honoraria from Pfizer Japan, Taiho Pharmaceutical, Chugai Pharmaceutical, Eli Lilly Japan, AstraZeneca, Boehringer Ingelheim Japan, Ono Pharmaceutical, Novartis Pharma, GlaxoSmithKline, and Bristol‐Myers Squibb. His institution has received research funding from Merck Serono, Pfizer Japan, Taiho Pharmaceutical, Eisai, Chugai Pharmaceutical, Eli Lilly Japan, AstraZeneca, Boehringer Ingelheim Japan, Ono Pharmaceutical, Novartis Pharma, Daiichi Sankyo, GlaxoSmithKline, Yakult Honsha, Quintiles, Astellas Pharma, and Bristol‐Myers Squibb. Dr Fumiyoshi Ohyanagi has received speakers’ bureau from AstraZeneca, Novartis pharma, Daiichi Sankyo, Ono Pharmaceutical Co., LTD, Boehringer Ingelheim, Chugai Pharmaceutical.,LTD, Eli Lilly Japan KK, Taiho Pharma, MSD, and Pfizer. Kuniko Sunami, MD, PhD; Satoru Kitazono, MD, PhD; Dr Keita Kudo; Yuichi Tambo, MD; and Atsushi Horiike, MD: None declared. Dr Yoshiro Nakahara has received lecture fee from AstraZeneca, Boehringer Ingelheim, Chugai Pharmaceutical co., LTD, Eli Lilly Japan KK, Ono Pharmaceutical Co., LTD, Taiho Pharma, MSD; grant from Bristol‐Myers Squibb, Takeda, and Ono Pharmaceutical Co., LTD. Dr Shintaro Kanda has received honoraria from AstraZeneca, Ono Pharmaceutical, and BMS. Noriko Yanagitani, MD, PhD has personal fees from Chugai, Taiho Pharma, MSD, Ono, Novartis, Byer, Boehringer, outside the submitted work. Dr Hidehito Horinouchi reports grants and personal fees from BMS, grants and personal fees from Novartis, grants from Astellas, grants and personal fees from Taiho, grants and personal fees from Chugai, personal fees from Lilly, grants and personal fees from Astra Zeneca, grants and personal fees from MSD, grants from Merck serono, grants from Genomic Health, outside the submitted work. Yutaka Fujiwara, MD reports grants from Abbvie and grant and personal fees from Astra Zeneca, grant and and personal fees from BMS, grants and personal fees from Chugai, grants and personal fees from Daichi‐Sankyo, personal fees from Eisai, personal fees from Eli Lilly, grants from Incyte, grants from Merck Serono, personal fees and grants from MSD, grants and personal fees from Novartis, personal fees from Ono, outside the submitted work. Hiroshi Nokihara, MD, PhD reports grants from Pfizer and grant and personal fees from Taiho Pharmaceutical, grant from Eisai, grants and personal fees from Chugai Pharma, grants and personal fees from Eli Lily, personal fees from Novartis, personal fees from Daiichi Sankyo, grants from Glaxo SmithKleine, grants from Quintiles, grants from Astellas Pharma, personal fees and grants from AstraZeneca, grants and personal fees from Boeringer Ingelheim, grants and personal fees from Ono, personal fees from BMS, grants from Regenration, personal fee from MSD, and grants from Abbie outside the submitted work. Noboru Yamamoto, MD, PhD reports grants from Chugai, Taiho, Eisai, Lily, Quintiles, Astellas, BMS, Novartis, Daiichi‐Sankyo, Pfizer, Boefhringer Ingelheim, Kyowa‐Hakko Kirin, Bayer, Ono, Takeda, Japan Pharma, MSD, Merck, and personal fees from Ono, Chugai, AstraZeneca, Pfizer, Lilly, BMS, Eisai, Otsuka, Takeda, Boehringer Ingerheim, and Cimic., outside the submitted work. Dr Makoto Nishio has received personal fees as honoraria from Pfizer Japan, Chugai Pharmaceutical, Eli Lilly Japan, Taiho Pharmaceutical, Nichirei Biosciences, Elekta, AstraZeneca, Sanofi, Bristol‐Myers Squibb and Ono Pharmaceutical and consulting fees from Novartis, Ono Pharmaceutical, Chugai Pharmaceutical, Eli Lilly Japan, Taiho Pharmaceutical, Daiichi Sankyo and Pfizer Japan. His institution has received research funding from Novartis, Ono Pharmaceutical, Chugai Pharmaceutical, Bristol‐Myers Squibb, Takeda Pharmaceutical, Daiichi Sankyo, Taiho Pharmaceutical, Eli Lilly Japan and Pfizer Japan. Dr Yuichiro Ohe reports grants and personal fees from ONO, grants and personal fees from BMS, grants and personal fees from AstraZeneca, grants and personal fees from Chugai, grants and personal fees from Lilly, grants and personal fees from Taiho, grants and personal fees from Takeda, grants and personal fees from Novartis, personal fees from Kissei grants and personal fees from Pfizer, grants and personal fees from MSD, grants from Jassen, grants from LOXO Oncology, grants and personal fees from Kyorin, grants from Dainippon‐Sumitomo, personal fees from Celtrion, personal fees from Kyowa Hakko Kirin, personal fees from Amgen, personal fees from Nippon Kayaku personal fees from Boehringer Ingelheim, personal fees from Bayer and grants from Ignyta, outside the submitted work. Dr Yukio Hosomi reports personal fees from AstraZeneca, personal fees from Eli Lilly Japan, personal fees from Taiho Pharmaceutical, personal fees from Chugai Pharmaceutical, personal fees from Ono Pharmaceutical, personal fees from Bristol‐Myers Squibb, personal fees from Kyowa Kirin, personal fees from CSL Behring, outside the submitted work. No other disclosures are reported.

## AUTHOR CONTRIBUTIONS

YO has contributed to the concept and design of the study and manuscript writing. YO, FO, KS, and YG have coordinated in each institution. All the authors have contributed to care the enrolled patients, and were involved in data analysis and interpretation, revision and approval of final version.

## ETHICAL APPROVAL

The study protocol was approved by the institutional review boards of all institutions (Clinical trial registration: UMIN000010736) and conducted in accordance with the Declaration of Helsinki and the Guidelines for Good Clinical Practice in Japan. Written informed consent was obtained from all participating patients.
